# Partial EMT in Squamous Cell Carcinoma: A Snapshot

**DOI:** 10.7150/ijbs.61566

**Published:** 2021-07-13

**Authors:** Chengcheng Liao, Qian Wang, Jiaxing An, Qian Long, Hui Wang, Meiling Xiang, Mingli Xiang, Yujie Zhao, Yulin Liu, Jianguo Liu, Xiaoyan Guan

**Affiliations:** 1 Department of Orthodontics II, Affiliated Stomatological Hospital of Zunyi Medical University, Zunyi 563000, China; 2 Oral Disease Research Key Laboratory of Guizhou Tertiary Institution, School of Stomatology, Zunyi Medical University, Zunyi 563006, China; 3 Department of Gastroenterology, Affiliated Hospital of Zunyi Medical University, Zunyi 563000, China; 4 Microbial Resources and Drug Development Key Laboratory of Guizhou Tertiary Institution, Life Sciences Institute, Zunyi Medical University, Zunyi 563006, China

**Keywords:** Partial EMT, Squamous Cell Carcinoma, FAT1, HIPPO, NOTCH, TGF-β, Collective migration, Stemness, Therapeutic resistance

## Abstract

In the process of cancer EMT, some subgroups of cancer cells simultaneously exhibit both mesenchymal and epithelial characteristics, a phenomenon termed partial EMT (pEMT). pEMT is a plastic state in which cells coexpress epithelial and mesenchymal markers. In squamous cell carcinoma (SCC), pEMT is regulated, and the phenotype is maintained via the HIPPO pathway, NOTCH pathway and TGF-β pathways and by microRNAs, lncRNAs and the cancer microenvironment (CME); thus, SCC exhibits aggressive tumorigenic properties and high stemness, which leads collective migration and therapy resistance. Few studies have reported therapeutic interventions to address cells that have undergone pEMT, and this approach may be an effective way to inhibit the plasticity, drug resistance and metastatic potential of SCC.

## Introduction

Epithelial-mesenchymal transition (EMT), which refers to the biological process through which epithelial cells transform into cells with a mesenchymal phenotype and plays an important role in cancer progression, embryonic development, inflammation, tissue reconstruction, and fibrosis 1. EMT endows tumors with malignant properties, including aggressive behaviors, metastasis, cancer stem cell (CSC) activity, and therapeutic resistance 2. EMT was historically considered a binary switch, and any partial state was regarded as a metastable state or transient snapshot acquired during the EMT process. Transcriptional and epigenetic characteristics determine the potential gene regulatory networks, transcription factors and signaling pathways that control these different EMT transition states 3. However, many studies have shown that cancer cells can stably acquire one or more partial EMT (pEMT) phenotypes, and that they can exhibi a mixture of epithelial and mesenchymal characteristics at the molecular and/or morphological level 4, 5, 6, 7, 8, 9, 10, 11. pEMT is also known as the hybrid epithelial/mesenchymal (E/M), intermediate EMT, intermediate mesenchymal, incomplete EMT, semimesenchymal, and EMT-like phenotype 12.

The pEMT phenotype plays an important role in the tumor progression, process of organ branching morphogenesis, diabetic kidney disease and wound healing 13, 14, 15, 16. Cells in the pEMT state show greater tumor-initiating potential, therapeutic resistance and apoptosis resistance than purely epithelial or mesenchymal cells 17, 18. Tumorigenesis depends on single cells in the pEMT state, and cannot be replicated by mixing epithelial and mesenchymal phenotype cells 19. Cells with a pEMT phenotype cells exhibit loss of apical-basal polarity and have better motility, while maintaining adhesion characteristics with neighboring cells and acquiring mesenchymal-like characteristics 20; thus, they can assemble and move together. If these cell clusters reach the bloodstream intact, circulating tumor cell clusters (CTCs) clusters that can migrate collectively are generated 18. Small numbers of CTCs have the highest plasticity, are believed to possess CSC characteristics and the ability to initiate tumors, can form new lesions and are fatal in tumor patients 21. These results indicate that the pEMT phenotype cells may be the most suitable for metastasis. The feedback loop between EMT and immunosuppression promotes tumor progression 22, 23, but how pEMT affects tumor immunity remains undiscussed 24.

Squamous cell carcinoma (SCC) accounts for most esophageal cancers (upper esophagus) 25, more than 90% of cervical cancers 26, 90% of head and neck cancers 27, 30% of non-small-cell lung cancers (NSCLCs) 28 and 20% of skin cancers 29. Head and neck squamous cell carcinoma (HNSCC), esophageal squamous cell carcinoma (ESCC), NSCLC and cutaneous squamous cell carcinoma (cSCC) are prone to metastasis, recurrence and invasion and have low 5-year survival rates 30, 31, 32, 33. HNSCC is prone to cervical lymph node and lung metastasis, and its 5-year survival rate is only 43% 32. The 5-year survival rate for esophageal cancer is less than 13% 30. NSCLC accounts for 85% of lung cancers; approximately 40% are unresectable, and the 5-year overall survival (OS) rate is approximately 15.9% 34, 35. NSCLC includes a variety of cancer types, such as lung adenocarcinoma (LUAD), lung squamous cell carcinoma (LUSC), and lung large cell carcinoma. The largest subgroups of NSCLC are LUAD and LUSC, which are significantly different at both the transcriptome and cell control network levels 36. However, many current NSCLC studies have not distinguished the difference between LUAD and LUSC. In this review, the description of NSCLC in this review is focused more on LUSC than on LUAD. Many types of SCC have a poor response to treatment and are prone to metastasis, and thus, they are life-threatening. Therapeutic targeting of the pEMT maintenance mechanism and phenotype seems to be the best strategy to overcome the poor prognosis of SCC.

## Markers of pEMT in SCC

pEMT is usually evidenced by simultaneous expression of epithelial and mesenchymal protein markers in a single cell 37. A detailed single-cell analysis of more than 6000 cells in 18 cases of HNSCC found that some of the cells showed pEMT properties; Vimentin and integrin-α5 were upregulated, although epithelial markers were not downregulated 38. Immunohistochemical detection of E-cadherin and Vimentin expression in 200 oral squamous cell carcinoma (OSCC) patients confirmed that evidence of non-EMT, pEMT, and full EMT was present among the clinical samples, accounting for 49.5% (99), 43.5% (87), and 7.5% (14) of cases, respectively 39. According to the expression of ESCC extracellular adhesion proteins, Vimentin and the adhesion and junction components Claudin1 and Claudin7, ESCC cells may be divided into the epithelial, full EMT and pEMT subgroups 40. In another study, according to the expression level and localization of E-cadherin, Vimentin and N-cadherin, approximately 43% of the ESCC primary tumors and 53% of ESCC metastatic lymph nodes were classified as having a pEMT phenotype 41. The pEMT phenotype can also be induced in SCC cell lines. In the p16-positive cervical carcinoma cell line CERV196, β-catenin levels are increased under epidermal growth factor (EGF) induction, while the expression levels of vimentin and E-cadherin change only slightly, which indicates a pEMT phenotype 42. In addition, overexpression of Bcl-2 in the OSCC cell line HSC-3 induces the expression of N-cadherin but cannot completely eliminate E-cadherin expression or induce increased expression of typical EMT-related transcription factors (EMT-TFs) 43.

Pastushenko et al. 44 divided the pEMT state into early and late stages based on the expression patterns of the tumor cell surface markers CD106, CD61, and CD51. Epithelial tumor cells express epithelial cell adhesion molecule (EpCAM), early pEMT is characterized by lack of EpCAM expression and a CD106^+/-^/CD51^-^/CD61^-^ phenotype, late pEMT status is characterized by a CD106^+/-^/CD51^+^/CD61^-^ phenotype, and full EMT tumor cells exhibit a CD106^+/-^/CD51^+^/CD61^+^ phenotype 20, 44. The pEMT subgroup in the SCC model established by Ievgenia et al. 45 is negative for CD106, CD61, and CD51 or expresses only CD106. Expression of epithelial markers such as E-cadherin and EpCAM is lost in the early stage of EMT, while keratin 5/8/14 expression is retained in pEMT and disappears completely in the later stage of EMT 44. N-cadherin and Vimentin are highly upregulated in the early hybrid state, and their levels are maintained in late EMT. The expression of fibroblast activation protein (FAP), cadherin 11, complexes that regulate transcription of the collagen XXIV gene (Col24a1), lysyl oxidase-like 1 (LOXL1), matrix metalloproteinase-19 (MMP19), platelet-derived growth factor receptor (PDGFR) a/b or paired related homeobox 1 (PRRX1) increases in late EMT 44.

## Regulatory mechanism of pEMT in SCC

pEMT may be a transient state in the EMT process, but the pEMT state can be stabilized or maintained through several mechanisms 46. pEMT is controlled by signaling pathways, TFs, epigenetic regulators, phenotype stabilizing factors (PSFs) and posttranslational modifications 47.

### pEMT-TFs in SCC

TFs bind to and inhibit the transcription of genes encoding adhesion junction and tight junction molecules, thereby triggering EMT. These TFs include Snail1/2, zinc finger E-box binding homeobox (ZEB) 1/2, Twist and lymphoid enhancer-binding factor 1 (LEF-1) 48. pEMT, which is characterized by Twist-induced expression of ZEB1, Vimentin and Podoplanin (PDPN) but without the absence of E-cadherin expression, mediates the acquisition of invasive characteristics by cSCC 49. In clinical samples of NSCLC, the frequency of Snail2 and E-cadherin expression seems to control EMT phenotypic plasticity. Specific expression patterns of E-cadherin and Snail2 distinguish NSCLC with different phenotypic characteristics and are related to prognosis 50. In the model of early pEMT in SCC established by Ievgenia et al. 45, pEMT is induced by the expression of ZEB1 and sex determining region Y-box2 (SOX2). Snail1, Twist and ZEB1/2 are highly upregulated in the early hybrid state, and their levels are maintained in late EMT 44. Another study suggested that early hybrid EMT is initiated by the expression of ZEB1, P63, Twist and LIM-protein 2 and that Smad2 promotes late EMT 51.

### FAT1/HIPPO pathway-mediated regulation of pEMT in SCC

FAT1 is a transmembrane protein involved in the regulation of EMT, cell growth and actin dynamics and plays a key role in tumorigenesis and development 52. FAT1 inhibits tumor progression by activating Hippo signaling 53. The Hippo core complex controls the transport of YAP1/TAZ proteins to the nucleus, and abnormal upregulation of YAP1/TAZ expression or their nuclear localization occurs in SCC, which promotes tumor progression and metastasis 54. FAT1 deletion promotes the acquisition of pEMT status in mouse and human SCC, thereby increasing tumor stemness and metastasis. Depletion of FAT1 functionally activates the CAMK2/CD44/SRC axis, promotes YAP1 nuclear translocation-mediated ZEB1 expression, and stimulates the mesenchymal state of SCC 36. Moreover, depletion of FAT1 inactivates EZH2 and promotes the expression of SOX2, thereby maintaining the epithelial state 45. The results of a recent study also showed that the ZIP4-miR-373-LATS2-ZEB1/YAP1-ITGA3 signaling axis has a significant impact on pancreatic cancer metastasis and pEMT phenotype acquisition 55.

### NOTCH/Jagged pathway-mediated regulation of pEMT in SCC

A ligand (Delta-like 1/3/4, Jagged-1/2) binds to a receptor (NOTCH1-4) to activate Notch signaling, thereby initiating the intercellular communication system 56. Ligand binding induces conformational changes in Notch, resulting in exposure of the S2 site, which is sequentially cleaved by the A Disintegrin and Metalloproteinase (ADAM) family of proteases and the γ-secretase complex, thereby releasing the Notch intracellular domain (NICD), which then translocates to the nucleus 57. In the nucleus, NICD binds to the transcription factor CBF-1/suppressor of hairless/Lag1 (CSL) and regulates gene expression 57. The Notch signaling pathway can not only activate cell proliferation and antagonize apoptosis, but it can also participate in crosstalk with a variety of TFs to promote the occurrence of EMT, thereby enhancing cell activity, invasion and metastasis in vivo 58.

Numb, which regulates the endocytosis of adhesion molecules (such as E-cadherin), is important for epithelial cell-cell and cell-matrix interactions 59. In addition, Numb inhibits Notch intercellular signaling and suppresses complete EMT by stabilizing the pEMT phenotype, which promotes mass migration of NSCLC cells 49. In addition, Numb is associated with poor survival rates and increased lung cancer aggressiveness 60. EMT induction in a given cell to increases the levels of Notch ligands and can activate Notch signaling in neighboring cells; in turn Notch-Delta and Notch-Jagged signaling then induces EMT 60. Notch/Jagged signaling but not Notch/Delta signaling can cause aggregation of pEMT cells and maintain the population of cells with a pEMT phenotype 60. In addition, the pEMT phenotype is strongly correlated with CSC attributes and increased Notch-Jagged signaling 60. Jagged signaling is often related to the maintenance of CSC populations 61, and Notch-Jagged signaling-induced stemness may be caused by the pEMT phenotype 60.

### TGF-β-mediated regulation of pEMT in SCC

Transforming growth factor β (TGF-β) is a main driver of EMT. TGF-β is a secreted cytokine that can regulate cell proliferation, migration and differentiation of many different types of cells 62. In the process of TGF-β-induced EMT, the key effectors are transcriptional inhibitors of E-cadherin, such as Snail1/2, ZEB1/2 and Twist 62. In scleroderma-affected skin, TGF-β-induced pEMT-like changes are characterized by the induction of Snail1 without loss of E-cadherin. Similarly, HaCaT cells (human skin keratinocytes) under continuous TGF-β stimulation exhibit pEMT characteristics 63, 64.

pEMT depends on the TGF-β pathway and is involved in lymph node metastasis in NSCLC patients 65. In addition, TGF-β causes proliferation arrest and changes in epithelial morphology in benign and malignant HaCaT cells. The epithelial connexin ZO-1 and E-cadherin are downregulated in response to TGF-β in benign and malignant HaCaT cells but do not induce mesenchymal markers, which suggests a pEMT response 66. In SCC cells (SiHa and FaDu), acidosis-induced TGF-β activation can promote pEMT and fatty acid metabolism 67. TGF-β/Smad participates in crosstalk with the Wnt, Notch, Hippo, Hedgehog, PI3K-Akt, NF-κB, and JAK/STAT signaling pathways 68. TGF-β drives Notch1-mediated EMT to generate ESCC tumor-initiating cells with high CD44 expression and inhibits Notch3 via ZEB1 expression, thereby preventing cell differentiation and allowing pEMT to progression 69.

Nuclear factor E2-related factor 2 (NRF2) can prevent adequate EMT during wound healing 70, activates pEMT and maximizes the presentation of the pEMT phenotype 71. TGF-β transcription activates P21, thereby stabilizing NRF2, which significantly promotes glutathione metabolism and reduces the effectiveness of SCC treatment 72.

### Epigenetic regulators of pEMT-TFs in SCC

Epigenetic regulatory factors (acetylation, methylation and noncoding RNAs) and posttranslational modifications (ubiquitination, sumoylation and phosphorylation) regulate the expression of EMT-TFs, thereby regulating pEMT and subsequent metastasis, stemness and therapeutic resistance 38.

miR-200 is considered a marker of cancer cells and a determinant of the epithelial phenotype 73. miR-200 directly targets the mRNA of the E-cadherin transcriptional repressor ZEB1/2 73. Activation of p53 downregulates Snail expression by inducing miR-34a/b/c gene expression 74. The miR-200/ZEB axis is driven by the miR-34/Snail axis to form a three-component stable loop; miR-200^high^/ZEB^low^, miR-200^low^/ZEB^high^, and miR-200^medium^/ZEB^medium^ cells have epithelial, full EMT and pEMT phenotypes, respectively 75. Moreover, Sukanta et al. 76 analyzed the cell fate transitions among epithelial, pEMT and mesenchymal states and confirmed that these transitions are mediated by the miR-200/ZEB mutual inhibition feedback loop, which is driven by the expression level of Snail. In addition to regulating E-cadherin and Snail, miR-200 and miR-34 also inhibit Jagged and Notch/Delta, respectively 77, 78, 79. These regulatory mechanisms of miR-200/miR-34 can affect the pEMT status in SCC.

miR-151a promotes the contact and barrier properties of endothelial cells and promotes endothelial cell movement and angiogenesis by inducing Snail2, which is frequently amplified in solid tumors, including lung tumors 80, 81, 82. As an oncomiR in the pathogenesis of NSCLC, miR-151a promotes tumor cell growth by regulating the expression of E-cadherin, Fibronectin and Slug, among others. In addition, as a direct target of miR-151a, E-cadherin can inhibit the migration of NSCLC cells and the transition to a mesenchymal-like cell phenotype, which suggests that the miR-151a-mediated induction of E-cadherin inhibition is the main mechanism by which miR-151a enhances pEMT in NSCLC 83.

Based on a comparison of pEMT cells with non-pEMT cells by single-cell sequencing, Snail2 is the only activated EMT-TF in pEMT cells 38. Similarly, in the three-dimensional Madin-Darby canine kidney (MDCK) tubule formation system, Snail2 has also been shown to be a key regulator of pEMT processes in vivo 84. Laminin subunit beta 3 (LAMB3) and PDPNs are thought to be pEMT markers and to be related to cancer metastasis. The expression of the lncRNA MYOSLID in HNSCC is closely related to that of Slug, LAMB3 and PDPN. In addition, knockout of MYOSLID significantly reduces the expression levels of Snail 2, LAMB3 and PDPN but has no effect on E-cadherin and Vimentin expression. The lncRNA MYOSLID promotes invasion and metastasis by regulating the pEMT process in HNSCC 85.

### Cancer microenvironment-mediated regulation of pEMT

The cancer microenvironment (CME) surrounding tumor cells contributes to the emergence, stabilization and regulation of the pEMT phenotype, thereby promoting tumor progression 86. The CME reflects the heterogeneity, spatial organization and complex fusion of tumor cells, fibroblasts, endothelial cells, immune cells and other mesenchymal cells in the surrounding extracellular matrix (ECM). Cancer-associated fibroblasts (CAFs) directly interact with cancer cells to promote pEMT 87, 88. The local distribution of CAFs in the CME differs significantly between patients with local relapse and those without relapse. While those with relapse accumulate more CAFs, cancer cells adjacent to CAFs express both E-cadherin and Vimentin 88, which is similar to the findings in the study by Wang et al. 89. pEMT cells are located at the primary tumor front in HNSCC and are near CAFs 38. The paracrine interaction between CAFs and malignant cells promotes the pEMT program at the tumor front in HNSCC and plays a potential role in tumor invasion 38. In cSCC and breast tumors, different EMT populations are distributed in different tumor areas and are related to specific microenvironments 44. With the development of EMT in tumor cells, the composition of different matrix components changes; most noticeably, the immune infiltration of monocytes and macrophages increases significantly, and the density of blood and lymphatic vessels increases 44. The reduction in macrophages in vivo increases the proportion of EpCAM^+^ tumor epithelial cells and cells in the early mixed EMT state and prevents further progression of EMT to a fully mesenchymal state 44.

Hypoxia is a powerful driving force for the disruption of normal tissue homeostasis and tumor-stromal interactions 90. The most characteristic hypoxia response pathway is mediated by hypoxia inducible factor-1 (HIF-1) 91. Hypoxic ESCC cells express high levels of HIF-1A and EIF5A2. The two-way regulation between HIF-1A and EIF5A2 plays an important role in ESCC metastasis, invasion, angiogenesis, and pEMT phenotypic coexpression of E-cadherin and Vimentin 92. In addition, periodic or intermittent hypoxia may stabilize the pEMT phenotype via HIF-1A stabilization and/or crosstalk between NRF2 and HIF-1A 93.

The extracellular pH of most solid tumors is acidic due to the high lactate production rate and poor perfusion. Acidosis induces genomic instability, promotes local tumor invasion and metastasis, and inhibits antitumor immunity and therapeutic resistance, thereby promoting tumor progression 94. Acidosis triggers the upregulation/activation of TGF-β2 in a TSP-1-dependent manner and promotes pEMT in SCC through TGF-βRI and subsequent phosphorylation of Smad2/3 69.

## pEMT and collective migration in SCC

Metastasis is a process by which localized cancer becomes a systemic disease; cancer spreads due to the migration of individual cells of the primary tumor. However, new evidence found in many types of cancers, such as breast cancer, lung cancer, and mesenchymal tumors, indicates that tumor metastasis can also occur via the spread of large, cohesive cell populations that accumulate in adjacent tissues 95. Collective migration does not require complete EMT. Cell clusters maintain intercellular connections and have some epithelial features; the acquisition of interstitial features allows the cells to migrate as a cluster. In contrast to the complete loss of cell-cell adhesion, which occurs during single-cell migration, during collective migration, cell clusters maintain cell-cell connections primarily through E-cadherin, gap junctions, and surface adhesion proteins of the immunoglobulin family 96. Collective migration is related to metastasis, and the clinical effect of collective tumor cell migration is worse than that of single-cell migration, as collective migration confers greater potential for metastasis and proliferation as well as higher therapeutic resistance 95, 97.

A higher pEMT score is related to HNSCC lymph node metastasis and higher lymph node staging 38, 98, and pEMT is associated with a greater metastasis rate of SCC cells 45, 88. In HNSCC, Snail induces Claudin-11-mediated Src activation and then suppresses RhoA activity at intercellular junctions through p190RhoGAP, maintains stable cell-cell contacts and induces collective migration 99. In addition, increased Src activity stabilizes E-cadherin-based connections and collective migration of HNSCC cells 100. In addition, Snail-mediated epithelial phenotype maintenance is important for the collective migration of SCC cells during EMT 99, 101, 102, 103, which suggests that the pEMT phenotype may affect the migration pattern in SCC.

Cells in migrating clusters are usually organized into two groups: leader cells and follower cells. Leader cells are responsible for sensing the microenvironment, generating traction to move other members of the group and reshaping the matrix through proteolysis to create a path through which the group can navigate 104. Fourteen differentially expressed mutations were found between the leader and follower subgroups isolated from NSCLC. The functional characteristics of the two phenotype-specific candidate mutations indicated that ARP3 enhances collective invasion by promoting the phenotype of leader cells and that KDM5B inhibits chain-like cooperative behavior in follower cells 105, in contrast to pEMT plasticity 106-107, supporting the relationship between pEMT, collective migration, and leader/follower cell genetic and phenotypic differences.

## pEMT and SCC stemness

EMT is associated with an increase in tumor stemness 108-109, 110. However, complete EMT may reduce the tumor initiation potential 111, 112, 113, 114. Some studies have reported that the stemness of CSC is most likely to be maintained in the pEMT state rather than in the pure epithelial or mesenchymal state 115, 116. Clustered cancer cells have been reported to have greater potential to become CSC compared with individual cancer cells^117^-^118^. A coupled EMT-stemness network showed that acquisition of the pEMT phenotype can increase tumor stemness 119. Moreover, research has shown that pEMT can greatly promote the stemness of SCC 120. At least two factors may lead to enhanced stem cell properties of pEMT cells: pEMT cells have the ability to self-renewal ability 109, 121-122 and can generate hybrid subgroups of epithelial and mesenchymal cells 109, 123-124. pEMT cells can be considered conceptually similar to adult stem cells in tissues, and this view is supported by the pEMT phenotype found in subpopulations of breast CSC 109, 125-126. However, stemness does not always monotonically increase during EMT. When cells begin to undergo EMT, stemness may initially increase, and when cells cross the pEMT threshold to acquire a complete mesenchymal phenotype, their stemness may decrease 109.

The mechanism by which pEMT regulates stemness in SCC is still largely unknown. However, some evidence has helped us understand the connection between pEMT and stemness. In NSCLC, TGF-β1 promotes the expression of CD133 in pEMT cells, which in turn leads to the conversion of non-stem cells to CSC 127. The OvoL family transcription factor Shavenbaby (Svb) is the downstream target of the Wnt and EGFR pathways, and this protein mediates their activity with regard to stem cell survival and proliferation. In addition, systemic steroid hormones produced by the ovaries regulate the conversion between Svb inhibitors and activators. Therefore, the Svb axis allows the internal integration of local signal cues and interorgan communication to regulate the proliferation and differentiation of stem cells and has a wide range of roles in adult stem cells and cancer stem cells 128. A recent study showed that the interaction of EMT-TFs (Snail, Zeb1/2) and the epithelial stabilizing factor Svb can regulate stemness and pEMT 129. In the SCC model established by Ievgenia et al.^45^, the expression of CD44 and SOX2 mediated by changes in the HIPPO pathway is an important mechanism for the emergence and maintenance of pEMT. However, CD44 and SOX2 are one of the key proteins that regulate tumor SCC stemness 130. Based on these studies, while pEMT promotes stemness in SCC, the maintenance of the pEMT phenotype may depend to a large extent on the promotion of tumor cell stemness by some pathways.

## pEMT and therapeutic resistance in SCC

NSCLC patients usually benefit from treatment with epidermal growth factor receptor (EGFR) tyrosine kinase inhibitors (TKIs), such as gefitinib and erlotinib. However, EGFR resistance is likely to occur and is at least partially mediated by EMT 131, 132, 133, 134, 135, 136. The frequency of Vimentin and E-cadherin coexpression in erlotinib-resistant NSCLC cell lines is significantly increased compared with that in the parental cell lines. In NSCLC-resistant cells, the pEMT phenotype, collective cell migration and increased stemness are associated with erlotinib resistance 137. CTCs isolated from NSCLC patients exhibit both PD-1 ligand 1 (PD-L1) positivity and the pEMT phenotype, which may represent the molecular background of immune escape in NSCLC 138. The pEMT phenotype may lead to unique and broad drug resistance to a variety of cancer therapies. Therapies targeting CSC in the pEMT state are expected to prevent metastasis and treatment resistance in OSCC 139. In an OSCC cell line, the epithelial cell subset (CD44^high^/EpCAM^high^/CD24^low^) is sensitive to cisplatin, paclitaxel and salinomycin; the mesenchymal cell subset (CD44^high^/EpCAM^low^/CD24^low^) is sensitive to cisplatin and salinomycin; and the pEMT subgroups (CD44^high^/EpCAM^low^/CD24^high^) are resistant to all three drugs 139.

Drug resistance is often associated with the EMT process and CSC. Increasing evidence shows that traditional therapies often fail to eradicate cancer cells that have been activated by EMT programs and that have become CSC, thereby allowing CSC-mediated clinical recurrence 140. Like EMT, the coexistence of pEMT and CSC is associated with poor prognosis and therapeutic resistance in cancer patients 115. Similar to normal stem cells, most CSC grow slowly, which is one of the reason why CSC are resistant to chemotherapy 141. It is reasonable to believe that pEMT cells acquire stronger drug resistance because of their stronger stemness. However, more direct and detailed studies are needed to determine the role of pEMT cells in the treatment of drug resistance.

## Conclusions

Through EMT studies on breast, pancreatic and ovarian cancer, the existence of pEMT was confirmed 142. pEMT is not a metastable transient state acquired during EMT but a stable phenotype. In addition, the functional role of pEMT may vary depending on the type of tumor, the state of spread, and the degree of metastasis and colonization 142. At present, the research on pEMT in SCC has just started, but some phenomena have been observed. Through pEMT, SCC cells acquire a stronger tumor initiation ability than that of epithelial and mesenchymal phenotype cells and undergo collective migration, thereby promoting invasion and metastasis into SCC patients' lymphatic and circulatory systems. After pEMT phenotype cells reach a suitable niche, the epithelial tumor phenotype can be completely or partially restored by mesenchymal-epithelial transition (MET) 96. A stochastic dynamics study, which used the dimension reduction approach of landscape (DRL) method to study the gene regulatory network interacting with metabolism and EMT, found a wide range of parameters that can produce four stable states, corresponding to epithelial (E), abnormal metabolism (A), pEMT (H) and mesenchymal (M) cell state. Further calculations quantified the transition path between these states and regarded it as a biological path. Cells tend to follow the sequence during EMT or MET. For EMT, cells in the E state need to enter the A state first , and then enter the M state, while for the MET, before the cells in the M state reaching the E state, cells are likely to enter the H state first.^143^. Therefore, tumor cells in the mesenchymal state can still transform into the pEMT phenotype when the microenvironment changes. The driving factors (internal and external) of pEMT in SCC cannot be fully determined. Changes in some signaling pathways (the HIPPO, NOTCH, and TGF-β pathways), EMT-TFs, the CME and noncoding RNAs can produce or/and maintain the pEMT phenotype in SCC. PSFs such as GRHL2, OVOL2 and miR-145 are also considered to be related to the maintenance of pEMT 144; however, the importance of PSFs for pEMT in SCC has not been reported.

Although we have noted the markers of each stage of EMT in SCC, which is very important for the study and separation of pEMT subgroups, there is no clear consensus on the current definition of pEMT based on the coexpression of selected mesenchymal and epithelial markers in SCC. Therefore, determining the model of epithelial and mesenchymal marker coexpression to standardize the characterization of pEMT remains a main challenge in this field. In addition, developing strategies to design treatments for pEMT subgroups is a direction worthy of consideration. The drug resistance and plasticity of pEMT subgroups, the tendency of SCC to metastasize and the poor prognosis of SCC patients with metastasis emphasize that traditional treatment methods cannot effectively overcome the poor prognosis conferred by pEMT.

## Figures and Tables

**Figure 1:**
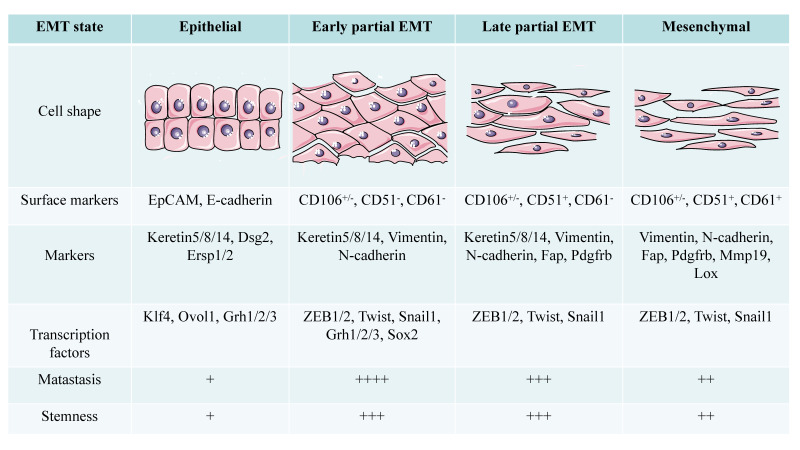
The conversion state of EMT shows different morphological and functional characteristics with different markers in SCC. Compared with cells in epithelial and mesenchymal state, partial EMT cells have stronger metastatic ability and stemness (+: low to ++++: high).

**Figure 2:**
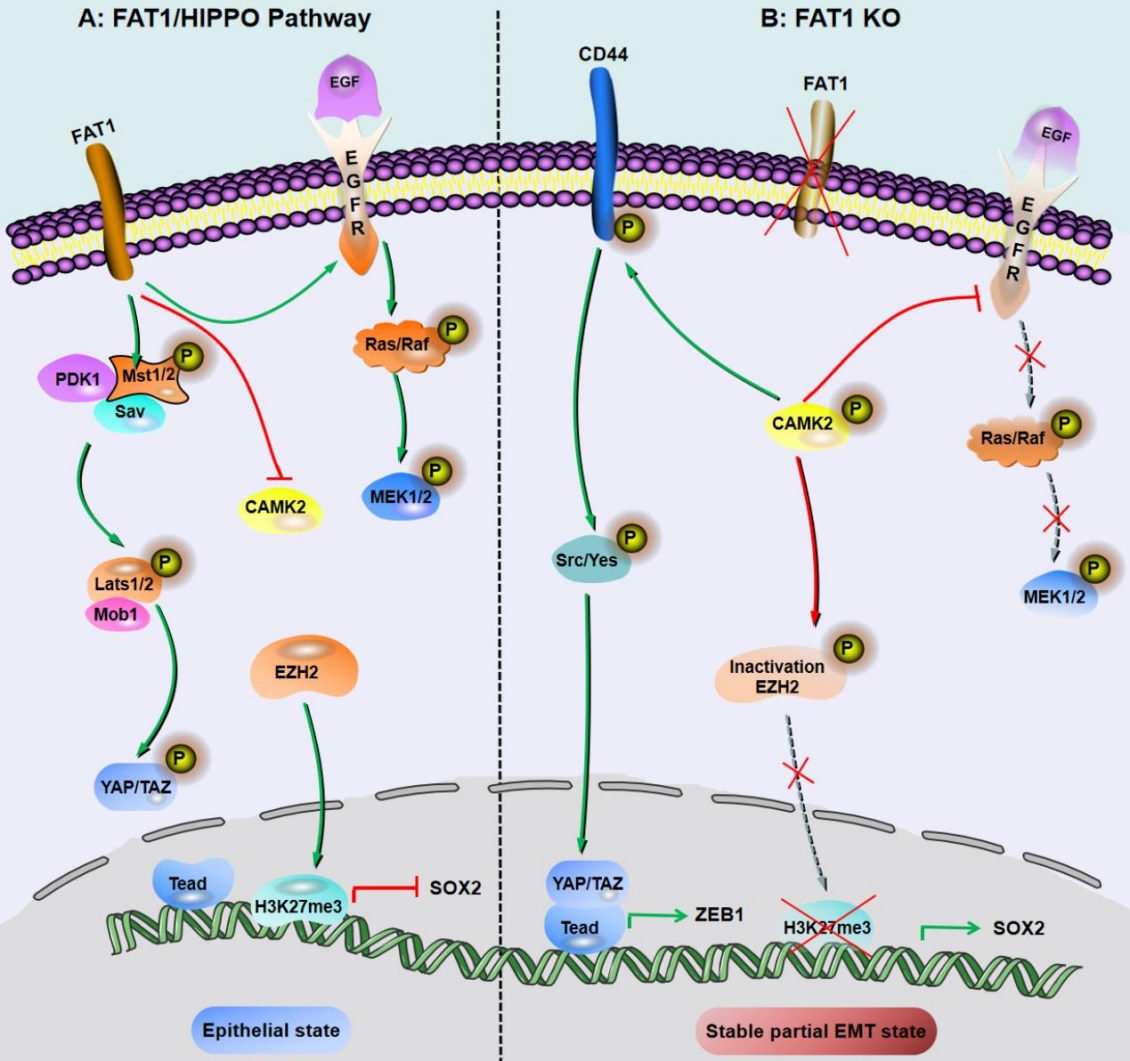
Inhibition of FAT1 can activate HIPPO-mediated partial EMT in SCC. A: Under the background of function of FAT1 and amplification or overexpression of EGFR, which is involved in the molecular mechanisms of SCC development. B: The loss of Fat1 accelerates tumor initiation and malignant progression, and promotes the partial EMT phenotype. The loss of FAT1 function activates the CAMK2-CD44-SRC axis, promotes YAP1 nuclear translocation and ZEB1 expression, and stimulates the mesenchymal state. This loss of function also inactivates EZH2 and promotes the expression of SOX2, thereby maintaining the epithelial state.

**Figure 3:**
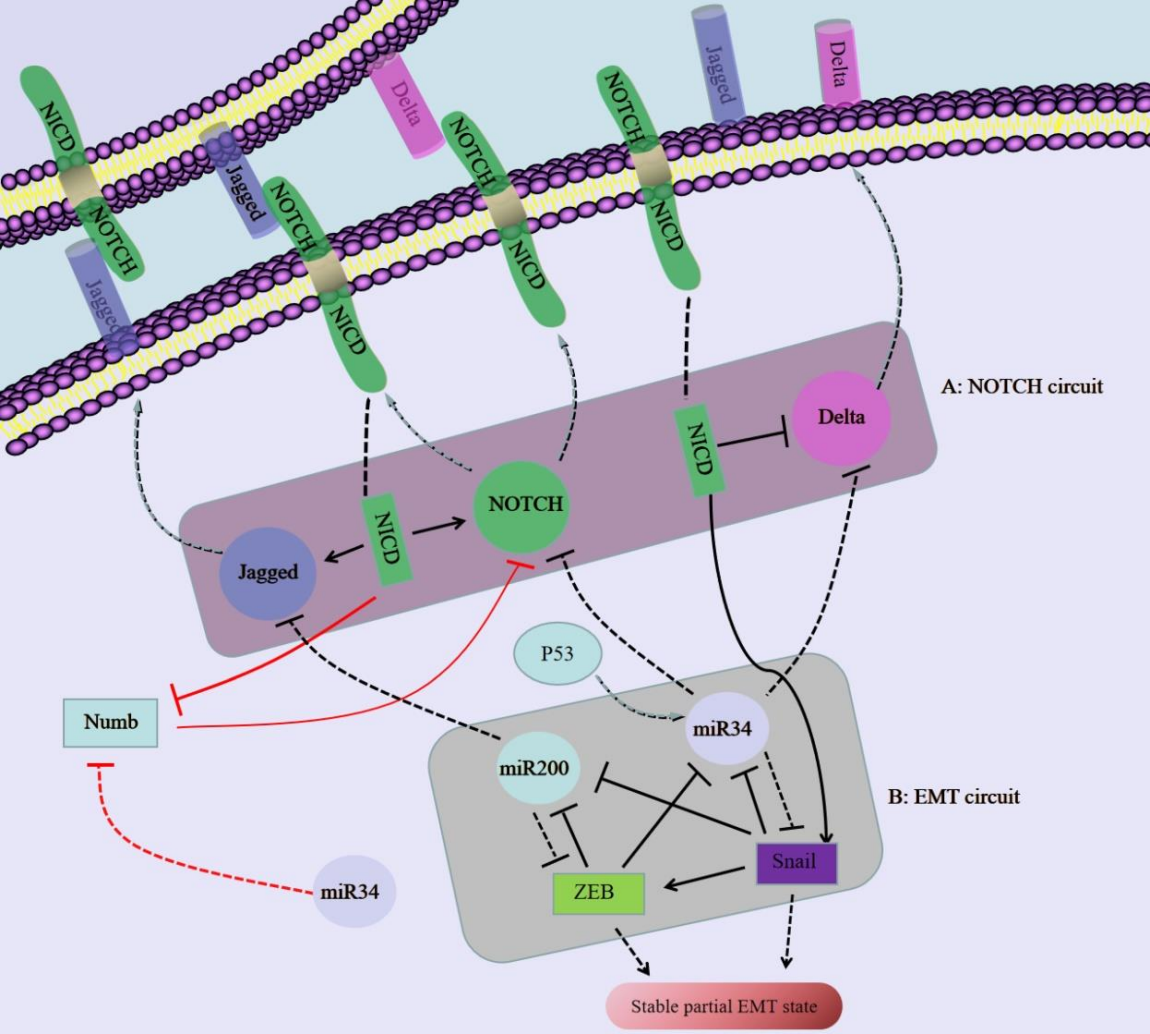
The regulatory network composed of NOTCH/Jagged and miR34/miR200 regulates the plasticity of partial EMT in SCC. A: Notch binds to an external ligand (Delta/jagged), resulting in the cleavage and release of Notch that produces NICD. NICD translocates to the nucleus, activates Notch and Jagged in transcription, and inhibits Delta at the same time. B: The EMT circuit contains miR-34, miR-200 and Snail, ZEB, which inhibit each other.

**Figure 4:**
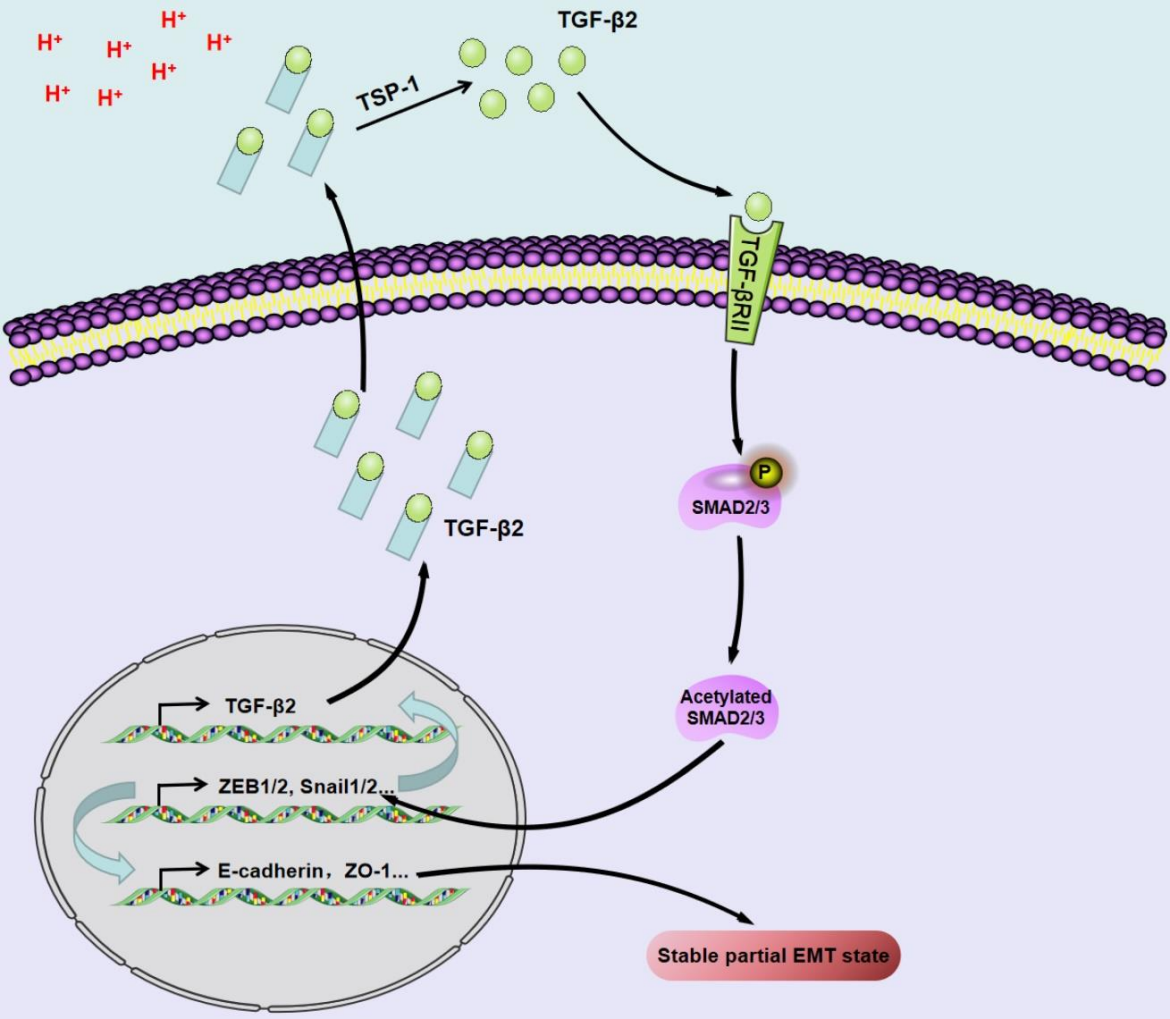
Acidosis triggers the upregulation/activation of TGF-β2 and the partial EMT phenotype in a Tsp-1-dependent manner in SCC.
